# The Microbiome-Gut-Brain Axis and Resilience to Developing Anxiety or Depression under Stress

**DOI:** 10.3390/microorganisms9040723

**Published:** 2021-03-31

**Authors:** Tracey Bear, Julie Dalziel, Jane Coad, Nicole Roy, Christine Butts, Pramod Gopal

**Affiliations:** 1 School of Food and Advanced Technology, Massey University, Palmerston North 4442, New Zealand; J.coad@massey.ac.nz; 2 The New Zealand Institute for Plant and Food Research Limited, Palmerston North 4410, New Zealand; Chrissie.Butts@plantandfood.co.nz (C.B.); pramod.gopal@plantandfood.co.nz (P.G.); 3 Riddet Institute, Massey University, Palmerston North 4442, New Zealand; julie.dalziel@agresearch.co.nz (J.D.); nicole.roy@otago.ac.nz (N.R.); 4 Smart Foods Innovation Centre of Excellence, AgResearch, Palmerston North 4442, New Zealand; 5 Department of Human Nutrition, Otago University, Dunedin 9016, New Zealand; 6 High-Value Nutrition National Science Challenge, Auckland 1145, New Zealand

**Keywords:** anxiety, depression, mood, gut microbiota, stress, probiotics, gut-inflammation, gut-permeability, enteric nervous system, vagus nerve

## Abstract

Episodes of depression and anxiety commonly follow the experience of stress, however not everyone who experiences stress develops a mood disorder. Individuals who are able to experience stress without a negative emotional effect are considered stress resilient. Stress-resilience (and its counterpart stress-susceptibility) are influenced by several psychological and biological factors, including the microbiome-gut-brain axis. Emerging research shows that the gut microbiota can influence mood, and that stress is an important variable in this relationship. Stress alters the gut microbiota and plausibly this could contribute to stress-related changes in mood. Most of the reported research has been conducted using animal models and demonstrates a relationship between gut microbiome and mood. The translational evidence from human clinical studies however is rather limited. In this review we examine the microbiome-gut-brain axis research in relation to stress resilience.

## 1. Introduction

Vulnerability to developing mood disorders such as anxiety disorders and depression depends on a mixture of genetic and environmental factors [1,2,3] and of the environmental factors, stress plays a significant role. Childhood adversity increases susceptibility to developing mood disorders later in life, and episodes of major depressive disorder are commonly preceded by psychosocial stress [4]. Not everyone who experiences stress develops a mood disorder [5]. Stress resilience is the ability to experience stressful events without the development of chronic elevated stress (psychological and/or biological) and associated changes in emotional behavior [6,7]. Stress susceptibility is related to psychological factors such as passive coping skills [7] and high emotional reactivity [8] but is also associated with biological factors such as hypo- or hyper- responsiveness of the stress response system (SRS), gonadal sex hormones, central and peripheral immune activation, and glucocorticoid resistance [6,7]. Interestingly, coping style is influenced by the level of biological stress response and associated neuroendocrine systems [7], and active coping under stress is increased with anti-depressant drugs [7]. This suggests that interventions which affect the biological side of stress resilience may be a useful adjunct to current treatments and preventative care.

The gut microbiome is a biological factor which is emerging as a possible influencer of stress resilience. The broad influence of the gut microbiota on human health, including psychiatric health, has begun to be realised and understood over the last decade [9]. The gut-brain axis is the bidirectional communication between the gut and the central nervous system that plays an important role in maintaining neural, hormonal and immunological homeostasis [10]. With emerging evidence showing that the gut microbiome can influence symptoms of depression and anxiety, the gut microbiome is now seen as a key component of this cross-talk between the gut and the brain and the term has been extended to microbiome-gut-brain axis (MGBA). Stress also alters the gut microbiota [11,12,13,14,15], and the effects of early life stress on the microbiota may extend to adulthood [16]. It is therefore plausible that changes in the gut microbiota due to stress at least partially mediate the onset of stress-related depressive or anxious episodes. This narrative review discusses research published on the interactions between stress and the MGBA and examines the evidence and potential mechanisms of how differences in stress-related changes in the gut microbiota may be associated with stress-resilience.

## 2. Stress and the Microbiome-Gut-Brain Axis

### 2.1. The Link between the Gut Microbiota and Behavior

The gut microbiota comprises around 0.2 kg of human body weight and has around the same number of cells as human eukaryotic cells (recently revised estimate) [17]. It also has around 150-fold more genes [18]. Interest in the gut microbiome has flourished in recent decades due to realization of its role in production of metabolic and endocrine products, and interactions with the host nervous and immune systems. The suggestion that the gut microbiota is linked with and may influence mood disorders began with the observation of a high co-morbidity of anxiety and depression disorders in people with gut disorders such as inflammatory bowel disease [19,20] and irritable bowel syndrome [19,20,21,22]. Correlational studies have shown that fecal microbiota composition in individuals with anxiety or depression (including those in remission) differs from that in healthy controls [23,24,25]. Women with a higher fecal *Prevotella* abundance experienced increased negative emotional response to viewing negative images, and lower brain activity in the hippocampus than those with a higher *Bacteroides* abundance [26]. Several studies in rodents have experimentally shown that the presence and composition of the gut microbiota can alter emotional behavior. In mice, gut infections or inflammation caused an increase in patterns of behavior thought to represent anxiety, including decreased exploration [27] and increased behavioral inhibition [28,29]. Germ free (GF) rats and mice (born and raised with no microbiota) show either increased or decreased anxiety and depressive-like behaviors compared with counterparts with specific pathogen-free (SPF) gut microbiota [30,31,32,33]. Some probiotics also show an effect on mood. Psychobiotics are probiotics which have been shown to “confer mental health benefits through interactions with commensal gut bacteria”. Not all probiotics or prebiotics are considered psychobiotics [34].

### 2.2. Inconsistencies and Problems with MGBA Research

The results of MGBA studies do not always agree, and the results from animal studies do not always translate well to human research. This has been a concern with MGBA research. Animal behavioral testing has limitations on how well it reflects anxiety- or depressive-like symptoms in humans, but there are methodological limitations with human studies due to heterogeneity of lifestyles and because it is difficult (or impossible) to collect certain biological samples such as colon microbiota and host tissues.

Probiotic supplementation has shown mixed effects on emotional behavior (refer to Table 1). There are several studies in rodents which show an amelioration of anxiety-like or depressive like behaviors following probiotic supplementation [35,36], inflammation-induced behavior changes [29,37,38,39], and stress-induced behavior changes [40,41]. Other studies have found no difference [42,43]. Translational research has shown variable results. No difference in mood was found in healthy adults [44] or in people with irritable bowel syndrome [45] following a probiotic supplement, but in other studies, depression scores were reduced in people with diagnosed depression [46] and healthy adults [47,48], or emotional reactivity was reduced [49]. Mixed results include a study where anxiety scores but not depression scores were reduced [50], and mood improved only in those who had low baseline mood [51].

Another interesting observation is that the direction of change in anxiety-like behaviors in GF rodents (compared with their SPF counterparts) seems to depend on the strain. Stress-sensitive strains (BALB/c mice and Fischer 344 rats) showed increased anxiety-like behaviors, in contrast to a decrease in anxiety-like behaviors in more resilient strains (NMRI mice, Swiss Webster mice and Wistar rats). Both increased and decreased anxiety-like behaviors occurred in mice when the gut microbiota were depleted with anti-microbial drugs [52,53,54]. A fecal transplant from an anxious-type mouse strain into a GF non-anxious strain caused an increase in anxiety behaviors [53]. The reverse was also observed, with a decrease in anxiety-like behavior in previously GF mice colonized from a non-anxious mouse strain [53].

**Figure 1:**
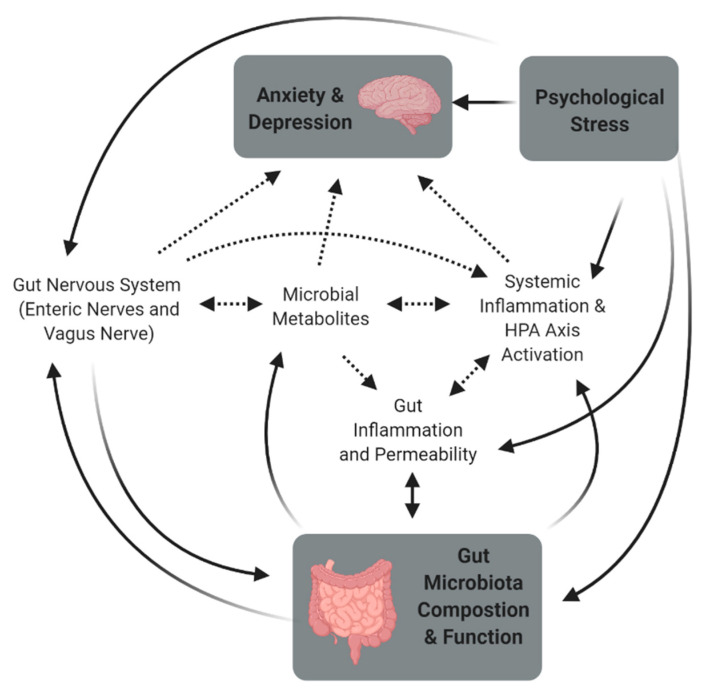
The proposed mechanisms of the microbiome-gut-brain axis (MGBA) are complex and intertwined. Emerging research shows that psychological stress interacts not only directly with the brain and mood, but also with many of the MGBA mechanisms thought to contribute to changes in mood with alteration of the gut microbiota. Solid lines indicate strong evidence of an effect, and dotted lines show proposed mechanisms with limited but emerging evidence. Abbreviations: HPA; Hypothalamic-Pituitary-Adrenal.

There are several reasons for the inconsistencies found in MGBA research. Probiotics effects are strain specific, and factors such as variation in survivability, ability to adhere to the gut mucosa, and their capacity to produce bioactive compounds [55]. The dose is also important and the efficacious dose may vary between probiotic strains. The variability in the effect on mood in GF rats suggest that it is the interactions of the microbiome with the host which is important, and that variations in host genotype/phenotype may be a key part of whether changes in the gut microbiota impact mood or not. Proposed mechanisms of the MGBA (Figure 1) are complicated, intertwined and bidirectional. Different hosts, with different life experiences (e.g., diet, stress, exercise), mean that the mechanisms for changes in mood could differ between animals and humans, animal strains, and possibly even individuals. Multiple mechanisms could also act in parallel.

### 2.3. Stress, the Gut Microbiota, and Behavior

There is interest developing in the interactions of stress with the MGBA. The gut microbiota composition can be altered under stress, as shown in rodent models of psychological stress [11,12,13,15,16,56,57,58,59,60,61,62,63,64]. Stress during pregnancy has also been shown to alter the gut microbiome structure in mouse offspring as well as the dams [65,66]. With the emerging evidence showing that alterations in the gut microbiota can influence mood, it seems plausible that stress-induced changes in the gut microbiota could (at least partially) mediate the development of chronic stress and/or anxiety and depression following a stressful event. Conversely, alleviation of the stress-induced changes in the gut microbiota and/or the physiological effects of this change may help to increase stress resilience.

Compositional changes in the gut microbiota following stress vary widely between studies and most of the evidence comes from rodent studies. Changes include a decrease in relative abundance of the genus *Lactobacillus* [15,56,58,67,68] and an increase in genera containing opportunistic pathogens such as *Odoribacter* [12,66], *Clostridium* [11,15], and *Mucisprillum* [13,66]. The genus *Bifidobacterium* has been shown to decrease under stress [15,63], and in one study was found to increase under stress in stress-resilient mice only [62]. The change in the gut microbiota may differ within different gut niches, for example restraint stress in male CD-1 mice caused a decrease in relative abundance of the genus *Lactobacillus* in the mucosa-associated microbiota but not the luminal microbiota [13].

The timeframe of the effect of stress on the gut microbiota varies. Similarly, recovery of the gut microbiota following stress seems to vary and changes can be persistent. Galley et al. [56] found differences in microbial beta diversity (which is comparison of changes in microbiota between samples rather than within samples) after only two hours of social stress in C57BL/6 mice, but a decrease in absolute abundance and relative abundance of *Lactobacillus* spp. after six days [56]. Infant rhesus monkeys had an altered gut microbiome three days following the stress of being separated from their mothers and placed instead in individual cages near other infant monkeys. Interestingly, five days post-separation, it was restored to the pre-separation composition [67]. In contrast, differences in fecal microbiota were found in rats seven weeks after maternal separation stress [16]. Bailey et al. [11] found that immediately following social stress, the gut microbiome of mice showed reduced diversity and richness, and clustered differently from the control group. However, after 15 h, the separation of the gut microbiota between groups was no longer as clear, with the stress group showing high variability, suggesting recovery of the gut microbiota following stress may vary between individuals.

Evidence from human studies is sparse. A study looking at the effect of diet and living conditions in (grounded) astronauts found that in fecal samples, the bacterial counts of *Bacteroides fragilis* subsp. *thetaiotaomicron* increased following interpersonal conflict in a confined living situation [68]. Prenatal stress in pregnant women was associated with persistently altered microbiota composition in their infants [69]. Increased relative abundances of *Proteobacteria* and a reduced relative abundances of lactic acid bacteria were found in the infants, which appear to be related to increased reports of gut problems [69].

The gut microbiota composition in humans or rodents is probably affected by a shift in the gut environment due to physiological changes in the gut under stress. Stress activates sympathetic pathways in the gut which regulate water absorption by gut epithelial cells; mucin production from goblet cells; gut permeability and inflammation (increasing mast cell degranulation and cytokine production) [70]. Increased gut motility and mucin secretion occur due to the mast cell degranulation [71,72]. Stress also causes slowed gastric emptying, but an overall decreased transit time, with increased distal colon motility [15,70]. Bacterial composition and function are also likely to be directly affected by circulating stress hormones [73]. Increases in concentrations of the catecholamines norepinephrine and epinephrine have been shown to increase the growth, virulence, and colonization of pathogenic bacteria [14,60,74,75]. Rodent stress models have shown increased colonization by *Citrobacter rodentium*, a colonic pathogen [60], and increased adherence and penetration of gut bacteria into mucosal cells [59]. Dexamethasone (an anti-inflammatory corticosteroid drug with similar actions to cortisol) administration in rats caused increased bacterial adherence and increased paracellular gut permeability (discussed in Section 3.2. in detail) [74].

## 3. Mechanisms Associated with Stress-Induced Changes in the Gut

### 3.1. Gut and Systemic Inflammation

Stress-induced gut inflammation could be a key mechanism for changes in emotional behavior under stress, with the gut microbiome function promoting or decreasing gut and systemic inflammation. Increased plasma inflammatory markers including interleukin (IL)-6 and tumor necrosis factor (TNF)-α have been found in people with anxiety [75] and depression [76,77] particularly in those who fail to respond to classical treatments [77]. Both depression and anxiety are more prevalent in people with inflammatory bowel disease (IBD), and are associated with more frequent IBD flare-ups and more severe IBD symptoms [78].

There is some evidence of the ability of the gut microbiome to promote or ameliorate systemic inflammation. Commensal microbiota seems to be associated with decreased inflammation, whereas potentially pathogenic bacteria are associated with inflammatory gut conditions [79,80,81]. In GF mice, the immune response to LPS is blunted [32,82,83], suggesting that the gut microbiota primes the immune system. Microglial activation, which causes neural inflammation, seems to be affected by the gut microbiota [37,82,83], possibly mediated by free fatty acid receptor 2 (FFAR2) in the gut, activated by short-chain fatty acids (SCFA) produced by the gut microbiota [84]. Both inflammatory and subclinical microbial gut infections, as well as chemically induced colitis increase anxiety and depression behavior or neurobiochemical markers in rodents [27,29,36,82,83,85]. Probiotic supplementation has been shown to attenuate pro-inflammatory markers or responses (*Lactobacillus helveticus* ns8 [41]; *Lactobacillus salivarius* UBL S22 [86]; *Lactobacillus farciminis* [87]; *Bifidobacterium infantis* 35624 [40,43]) and commercial mix VSL#3 [37]) although not always (*Lactobacillus rhamnosus* JB-1 [44]; *Bifidobacterium longum* NCC3001 and *L. rhamnosus* NCC4007 [29]). The probiotic *L. salivarius* UBL S22 also decreased the total fecal *Escherichia coli* count [86], a bacteria linked with gut inflammation [88].

The stress-induced gut microbiota composition, with decreased commensal microbiota and increased opportunistic pathogens, is likely to be inflammatory, but whether it is the cause of stress-induced gut inflammation and whether it mediates emotional behavior is unknown. Stress-induced inflammation also occurs directly via the sympathetic nervous system innervation of lymphoid organs [70] and activation of mast cells [72,73,89] and dendritic cells [90], however antibiotic administration in mice during stress prevented an increase in pro-inflammatory markers, suggesting a key role of the gut microbiota [11]. The probiotic *L. rhamnosus* JB-1 was also able to ameliorate stress-induced dendritic cell activation as well as reduce the effects of stress-related changes in anxiety-like behavior [90].

Seven days of repeated restraint stress in mice increased the immune response to a colonic pathogen challenge. A fecal transplant from the stressed mice into GF mice caused an increased inflammatory response to colonic pathogen (*Citrobacter rodentium*) in the GF mice, compared with a fecal transplant from non-stressed mice [63]. Whether the fecal microbiota itself or other molecules within the feces caused the increased immune response is unclear, but it is likely due to an interaction between the gut microbiota and the enteric immune system. The gut microbiota plays a fundamental role in the function and maturation of the gut immune system, including CD4 cells [91]. Susceptibility to colonic inflammation has previously been shown to increase following stress due to sensitization of CD4(+) lymphocytes, with the increased susceptibility able to be transferred to other rats with intravenous transfer of the CD4(+) lymphocytes [92].

### 3.2. Gut Permeability

Increased gut permeability was found in over 40 % of people with depression in one study [89], and may be a cause of increased systemic inflammation [93]. Gram-negative bacteria, such as *Proteobacteria* (including *E. coli*), have endotoxic lipopolysaccharide (LPS) chains on their outer cell wall, and with increased gut permeability, translocation of LPS from the gut lumen into the body occurs. LPS interacts with immune cells and induces the expression of several inflammatory molecules such as pro-inflammatory cytokines, nitric oxide, and eicosanoids, which are also found in those with depression [78,94,95]. Intravenous administration of LPS has been shown to increase anxiety and depression behaviors in people [96] and mice [84], and can induce neuroinflammation, causing microglial cells to become activated [97,98]. Evidence for increased gut permeability and bacterial translocation in people with depression has also been found, with increased concentrations of serum IgA and IgM against LPS [99], and associated increased activation of inflammation, oxidative and nitrosative stress pathways [93].

Increased gut permeability occurs under stress [87,94,95,100,101,102], with decreased expression of tight junction proteins [94] and an increase in translocation of large antigenic molecules [95,100,101]. The release of antigens triggers the inflammatory mechanisms such as activation of CD4+ cells resulting in mast cell mast cell degranulation, neutrophil infiltration and increased cytokine IFN-γ [94,102]. Increased gut permeability to antigenic molecules is likely to contribute to colonic inflammation due to reactivation of sensitized CD4(+) cells [103]. Whether increased gut permeability following stress occurs due to physiological reasons or because of a change in the gut microbiota is unclear. The increased permeability can be induced by dexamethasone or eliminated by adrenalectomy or glucocorticoid receptor blockade, suggesting physiological mechanisms [101]. However, the evidence does suggest that promotion of the growth of commensal bacteria with a decrease in LPS-producing bacteria is likely to alleviate stress-induced increases in gut permeability and associated pro-inflammatory immune activation. Dysbiosis, including a decrease in *Bifidobacterium* and an increase in LPS-producing bacteria has been associated with increased gut permeability [104], and probiotic supplementation (*L. farciminis* [87]; mix of *L. rhamnosus* R0011 and *L. helveticus* R0052 [59]; *L. paracasei* NCC2461 [105]) can attenuate stress-induced gut permeability in rodents. Whether supporting and promoting the growth of commensal bacteria under stress increases psychological resilience is the next level of research needed.

### 3.3. Dysbiosis and Hypothalamic-Pituitary-Adrenal Axis Dysfunction

A dysfunctional stress response and hypothalamic-pituitary-adrenal (HPA) axis may be a contributor to the development of both anxiety and depression [106]. The gut microbiome may also influence the stress response. The markers of the HPA axis such as corticosterone have been shown to be altered in GF mice compared with SPF counterparts [31,32,33,107]. Blunted corticosterone responses have also been found in adult rats exposed to early life stress, and the corticosterone concentrations negatively correlated to fecal *Akkermansia* and *Rikenella* [108,109]. A fecal transplant from depressed people into GF rats caused an increase in the rats’ corticosterone response to acute stress alongside increased depression-like and anxiety-like behaviors [110].

How the gut microbiota affects the stress response is uncertain. Immune activation activates the HPA axis [111,112,113], providing an indirect mode of effect of the gut microbiota on the stress response system. It is known that some bacteria produce catecholamines including norepinephrine, epinephrine and dopamine [114,115,116,117,118,119,120]. This is unlikely to cause a direct increase in concentrations of systemic catecholamines, because GF rats have typically been found with increased HPA-axis markers compared to rats with the normal gut microbiota [31,32,33,107]. It is plausible however that microbially produced catecholamines could contribute to baseline concentrations, and therefore HPA-axis programming in early life. Early life dysbiosis could therefore provide artificially high or low basal catecholamine concentrations and cause dysfunctional programming. This has not been tested.

The balance between pathogenic and commensal bacteria in the gut is likely to be important. Elevated plasma ACTH and corticosterone concentrations in GF rats were able to be prevented by the colonization of the GF mice at an early age with *B. infantis* or a mutant strain of *E. coli* lacking the translocated intimin receptor gene, neither of which are internalized into the gut epithelial cells. Wild-type *E. coli*, which do get internalized, did not prevent the heightened stress response [107]. Probiotic supplementation during or following stress has been shown to reduce corticosterone in stressed animals (*L. rhamnosus* JB-1 [36], *L. farciminis* [87], *L. helveticus* ns8 [41], mix of *L. rhamnosus* R0011 and *L. helveticus* R0052 [59] or monoassociation with *B. infantis* [107] or people (probiotic *L. helveticus* R0052 and *B. longum* R0175) [47], but *B. infantis* 35624 did not affect corticosterone in non-stressed animals [43]. However, no changes in systemic corticosterone concentrations were found following stress-induced changes in the gut microbiota and behavior in mice [58], or with amelioration of stress-induced behavioral changes in rats following supplementation with *B. infantis* 35624 [40]. Interestingly, a synbiotic supplement (*L. rhamnosus* GG + polydextrose and galactooligosaccharide) increased plasma corticosterone concentration in rats following acute stress compared to that of control animals, whereas the same probiotic and prebiotic mix given separately did not [64].

The HPA axis dysregulation in people with depression is in itself not a straightforward relationship, with both high and low levels of cortisol found, as well as other dysfunction such as delayed return to baseline following acute stress and glucocorticoid resistance. With the gut microbiota able to increase HPA axis activation, stress-induced dysbiosis may increase the physiological stress response higher than that which is required to deal with the stressor effectively. Whether the gut microbiome moderates stress-induced increases in HPA activation in a meaningful way, and whether this affects emotional behavior is unclear. More research is needed in this area.

### 3.4. Metabolites

Microbially derived metabolites include SCFAs, bile acids, choline and phenolic metabolites, indole derivatives, vitamins, polyamines, and lipids [121]. The metabolites are primary secreted signaling molecules (influenced by host-derived signaling molecules) which cross-talk with other microbes, and the host immune system, or secondary metabolites (produced through the metabolism of food, non-food ingested compounds, such as medication, and metabolites from other microbes).

SCFAs activate several receptors in the gut which have been shown to reduce colonic inflammation [122,123] and microglial neuroinflammation [84]. These receptors can also increase gut epithelial cell barrier integrity by increasing the expression of tight junctions [reviewed in [124]]. There is some evidence of altered SCFA production with depression and stress, although the direction of change is conflicting. No difference in SCFA concentrations was found in the fecal samples of people with depression compared with controls in two studies [110,125]. When fecal samples from depressed people were transplanted into mice, an increase in fecal acetate and total SCFA concentrations was found along with increases in depression-like behavior [110]. Prebiotic supplementation in mice increased SCFA concentrations many of which were negatively correlated with depression-like and anxiety-like behaviors [61].

A decrease in fecal acetate and butyrate, as well as SCFA-producing bacteria, occurred in mice following psychosocial stress, and this was associated with an increase in gut inflammation [126]. A similar study found an increase in cecal acetate, a decrease in propionate, butyrate and valerate, but no change in branched-chain fatty acids. No increase in systemic LPS was found, despite an increase in gut permeability to FITC-dextran, but this was likely due to the colonic mucus layer being unaffected. Interestingly, only minor changes in the gut microbiota composition at the family and genus level were observed following the stress intervention [127]. Functional changes in the gut microbiota have also been seen in mice following stress. KEGG analysis of 16S RNA marker genes in fecal samples predicted reduced pathways for the synthesis and metabolism of neurotransmitter precursors tyrosine and tryptophan, and SCFAs. This finding was positively associated with reduced exploration and sociability in the mice [128]. In contrast, children who had increased self-reported stress showed increased fecal SCFAs (butyrate, valerate, isovalerate and isovalerate), but no increased gut inflammation (based on fecal calprotectin concentrations) was found [129]. A possible explanation for the increased fecal SCFA is stress-induced decrease in gut transit time rather than a change in gut microbiota fermentation. Hair cortisol concentrations (a measure of long-term stress) in the children were not related to SCFAs, but heart rate variability was associated with decreased valerate [129]. Heart rate variability is a proxy for parasympathetic nervous system activation [130], which affects gut transit time [70]. Gut motility likely has bidirectional interactions with SCFAs and microbial composition. Stress induced dysmotility was able to be reversed in vitro with the application of either propionate or *L. rhamnosus* JB-1 [131].

Supplementation of an SCFA mixture in healthy adult men (174.2 mmol acetate, 13.3 mmol propionate, and 52.4 mmol butyrate), administered daily via the colon, reduced acute corticosterone response to acute stress, and increased serum SCFAs [132]. This result was not, however, associated with a change in subjective mood ratings [132]. In male mice, daily oral supplementation of the SCFAs (67.5 mmol acetate, 25 mmol propionate, and 40 mmol butyrate) decreased stress-related increases in anxiety-like behaviors in the open field test, and increased sucrose preference and decreased urine sniffing, both markers of depression-like behavior. The SCFA supplement was associated with changes in gene expression in the brain related to dopamine receptors, part of the mesolimbic reward pathway which can be altered in depression [127]. Cecal SCFAs also differ between stress-sensitive WKY rat strain compared with the stress-resilient Sprague Dawley rat strain [133]. Anti-inflammatory effects of an increase in SCFA in some of the populations studied could be contributing to stress resilience.

The gut microbiome is a source of vitamins, including vitamin K and B vitamins niacin, biotin, riboflavin, folate and pyroxidine [134,135,136,137,138]. Serum folate (B9) and pyroxidine (B6) are lower in those with depression or an increased risk of depression [139,140,141,142]. Micronutrients may affect depression risk via effects on the production and activity of monoamine neurotransmitters such as serotonin [143,144,145,146,147,148], alterations to the HPA system [149], glutamatergic signaling [149], or inflammatory and oxidative stress [149,150]. They also play a role in the gut, for example, niacin is anti-inflammatory in the gut due to activation of the Gpr109a receptor, the same receptor that is activated by the SCFA butyrate [122]. Folate and biotin are also immunomodulatory [151,152]. Pyroxidine (B6), is an essential co-factor for several enzymes in the kynurenine pathway [153], and a deficiency increases levels of xanthurenate, a kynurenine metabolite which is an antagonist for glutamate receptors. GF rats show increased susceptibility to developing B6 deficiency [136], and an accumulation of xanthurenate [154]. Changes in the gut microbiota could alter the available concentrations of microbially produced vitamins, plausibly contributing to immune and metabolic pathway changes which are related to mood.

Up to 95% of the neurotransmitter serotonin, which has a well-known link to anxiety and depression [155] is produced endogenously in the gut mucosa [156], and secretion of serotonin from enterochromaffin cells is influenced by microbial metabolites [157,158,159,160]. It is also produced by the gut microbiota, along with several other neurotransmitters including dopamine, gamma aminobutyric acid (GABA), acetylcholine and norepinephrine [115,116,117,118,119,120]. Neurotransmitter levels and turnover in the brain differ in GF mice [32,33,161] and reduced levels of circulating GABA and serotonin have been found in GF rats [162,163]. Whether alterations of these metabolites in the gut (endogenous and microbially produced) are related to those in the brain is uncertain, but there is some evidence that they can cross the blood–brain barrier [164], of which the permeability is in itself affected by the gut microbiota [165]. Whether gut metabolites can directly reach the brain or not, they can affect the gastric environment and neural signaling. For example, GABA and acetylcholine are immunomodulatory [166,167], and GABA and serotonin affect gastric motility and acid secretion via enteric neurons [160,161]. Whether stress-related changes in the gut microbiota alter the concentration of microbially produced vitamins or neurotransmitters in the gut is unknown.

Changes in metabolic pathways are another way that neurotransmitters are altered. Chronic stress in mice caused a decrease in the genus *Lactobacillus*, and a correlated increase in depression-like behavior. Supplementation with the probiotic *Lactobacillus reuteri* ATCC 23272 decreased the depression-like behavior, seemingly via the production of H_2_O_2_ which inhibits the enzyme indoleamine 2,3-dioxygenase 1 (IDO1) and restores the balance of serotonin/kynurenine pathways [15]. IDO1 is activated by inflammation and LPS [168].

### 3.5. Gut Nervous System—Enteric Nerves and Vagus Nerve

Autonomic nervous system dysfunction, with increased sympathetic tone and decreased parasympathetic (vagal) tone, is proposed to be a contributing factor in the development of depression [169]. People with depression commonly show decreased heart rate variability, a measure of high sympathetic activity [170], and vagal nerve stimulation may be effective for treatment-resistant depression, e.g., [171], although more research is needed [172].

The vagus nerve is linked with both the HPA axis and the immune system. Afferent fibers of the vagus nerve innervate the nucleus of the solitary tract, a brain region that directly regulates the HPA axis. Vagal nerve stimulation therapy can normalize HPA activity [169]. Secondary fibers also innervate brain regions responsible for emotional regulation [169,173]. Vagal sensory endings have receptors for cytokines and relay information about thoracic and gut inflammation to the brain [173]. In response, efferent fibers of the vagus nerve influence inflammation via cholinergic signaling, which inhibits cytokine release from LPS-stimulated macrophages. This negative feedback effect is known as the vagal-immune reflex. Low vagal tone is thought to promote systemic inflammation [174,175]. There may also be an influence on neuroinflammation via receptors located on microglia and astrocytes [169].

Gut infection or systemic immune challenge with the bacterial endotoxin LPS caused vagal ganglia activation (shown by FOS immunoreactivity) [27,82]. Associated sickness and anxiety-like behavior were able to be alleviated with vagotomy [39,88,176], suggesting that the vagal signaling can mediate the development of emotional behaviors. Increased vagal activation also occurs with probiotic supplementation (*Lactobacillus johnsonii* La1 [177]; *B. Infantis* [107]), and vagotomy prevented the restorative effect of probiotics on anxiety (*B. longum* NCC3001 [39]; *L. rhamnosus* JB-1 [36]). Early life stress in rats increased the cholinergic secretory response of enteric nerves to stimulation [59]. While there is strong evidence for the vagus nerve being a key mediator in the gut-brain axis, some studies have found changes in emotional behavior in mice due to gut infection [29] or anti-microbial treatment [53] despite a vagotomy procedure [29,53]. The vagus nerve may just be one mechanism of transmitting infection information to the brain.

## 4. Early Life Programming

Childhood adversity and stressful life events are both strongly linked with an increased risk of developing depression [4,5,178]. Early life stress in rodents (during the neonatal period) can cause increased anxiety-like and depressive-like behaviors in adulthood [64,179,180,181]. Stress during adolescence in mice also caused increased anxiety-like behaviors in adulthood [182]. In contrast, a safe and reliable childhood with strong maternal/caregiver attachment decreases the risk of anxiety and depression later in life, even in those with a higher genetic risk [7,183]. The stress response system (SRS) is the biological response to both psychological and physiological (such as illness, injury) stressors. Both a hyper and hypo-responsive SRS are linked with mood disorders [176,184,185]. The SRS is functionally and epigenetically programmed in early life to match the individual’s phenotype to their environment [178,186,187]. Similar epigenetic programming occurs in early life for the immune system. Early life stress causes an inflammatory immune phenotype characterized by increased pro-inflammatory cytokines (IL-1β, IL-6, TNF-α) which are also associated with depression [16,188]. Whether the early life stress causes independent epigenetic changes to the immune system or is mediated through epigenetic modulation of the SRS is debated [188]. The two systems are interlinked [111,112,113]; early exposure to high cortisol levels can cause immune dysfunction later in life [187].

The gut microbiota may play a role in this early life programming. The biological response to physiological and psychological stressors are similar, and it is likely that exposure to the increases in inflammation and alterations in HPA-axis signalling due to stress-induced dysbiosis, maybe a significant part of the environmental signalling which causes epigenetic programming. Injections of LPS in rats in the neonatal period [178] and in early adolescence [189] caused increased anxiety-like behaviors later in life, and changes in GABA, corticotropin-releasing hormone (CRH) and glucocorticoid receptors in the hippocampus and hypothalamus in [178]. Maternal Separation Stress (MSS) has been shown to increase gut permeability [59], and therefore systemic LPS exposure. Whether interventions to manipulate the gut microbiota in early life can prevent or reduce the effects of early life stress is still being elucidated.

Probiotic supplementation with *L. rhamnosus* strain R0011 (95%) and *L. helveticus* strain R0052 (5%) during the separation period of MSS was able to prevent the stress-induced increase in serum corticosterone, adherence and penetration of bacteria into mucosal cells, and increase in gut permeability [59]. Additionally, increases in anxiety-like behavior and changes in gene expression in rats following MSS were able to be ameliorated by dietary supplementation of the probiotic *L. rhamnosus* GG alone or in combination with prebiotics polydextrose and galactooligosaccharide [64]. In contrast, supplementation of rats with omega-3 fatty acids eicosapentaenoic acid (EPA) and docosahexaenoic acid (DPA) following MSS restored the gut microbiota [108] and prevented higher levels of corticosterone in response to stress [109], but caused reduced anxiety and increased cognitive performance in the non-stressed rats only, with no difference in behavior in the stressed rats [109]. These findings may be due to the intervention being after rather than during the stress period, the type of intervention, or an indication that the gut microbiota are not the key mediator of the behavioral effects of early life stress.

## 5. Therefore, Could the Gut Microbiota Be Key in Stress-Resilience?

The evidence for stress-induced changes in the gut microbiota being a mechanism rather than a covariate of stress-induced changes in mood is limited and sometimes conflicting, but has plausible mechanisms. If stress-induced changes in the gut microbiota do reduce stress resilience then differences between individuals who are stress-resilient and stress-sensitive and/or correlations of the gut microbiota with mood symptoms should be observed. There is some evidence to support this. Studies investigating links between stress, the gut microbiome and mood are summarized in Table 2.

A comparison of stress-resilient mice showed increased *Bifidobacterium* spp. in the stress-resilient mice compared with control mice or stress-sensitive mice [62]. Some strains of *Bifidobacterium* are considered psychobiotic so it seems straightforward that an increase in their abundance in some of the rats could promote stress resilience. However, the mechanism for how an increase in *Bifidobacterium* would occur due to stress was unclear.

Other studies did not compare stress-resilient and stress-sensitive mice, but found some correlations between gut microbiota and behavior. Marin et al. [15] found decreased *Lactobacillus* in male mice following chronic mild stress and a positive correlation between *Lactobacillus* and escape behaviors (active swimming) in the forced swim test [15]. Bansgaard Bendtsen et al. [12] found that in female BALB/c mice exposed to two weeks of grid floor stress, the cecal microbiota differed from that of the control group and was correlated with behavior. The time spent in the dark compartment of the light/dark box test (considered as anxiety-like behavior) was positively correlated with *Ruminococcaceae* spp., which was also negatively correlated with pro-inflammatory cytokine interleukin-2. Time spent in the closed arm of the elevated plus maze (also considered anxiety-like behavior) was negatively correlated with the genus *Butyricicoccus* (a butyrate producer). In the same study, risk assessment behavior (two paws placed in open arms and then retracted) were positively correlated with *Lachnospiraceae*, and in the control group only, the relative abundance of *Bacterioides* (a major propionate producer) positively correlated with the number of immobility episodes in the tail suspension test [12]. Likewise, comparison of stress-sensitive WKY rats and stress-resilient Sprague Dawley rats under acute stress showed an increase in relative abundance of cecal *Lactococcus*, a lactic acid producer. *Lactococcus* was positively correlated with brain and plasma lipid metabolites [133].

The evidence for specific microbiota relating to behavior is sparse but suggests that changes in SCFAs and inflammation may be key mechanisms. These could be linked due to SCFAs being immunodulatory. Stress-induced decreases in the genus *Lactobacillus* have been found in several animal stress studies [11,15,56,57,58,59,60,61,62,63,64,65,66,67,68]. It is plausible that individual differences in stress-induced decrease in *Lactobacillus* could be the difference between an in individual being stress-resilience or stress-sensitive. Marin et al. [15] found that kynurenine was increased in stressor-exposed mice alongside the lowered *Lactobacillus* and that while a supplement of the probiotic *L. reuteri* ameliorated stress-induced behavior, it did not work if L-kynureine was also supplemented alongside. They found that in vitro, a reactive oxygen species produced by *Lactobacillus* inhibited the enzyme indoleamine 2,3-Dioxygenase 1 (IDO1), a key enzyme which allows tryptophan to be converted to kynurenine. IDO1 is also activated by inflammation and therefore could be a mechanism

Not all studies have found associations between microbiota composition and behavior. Tsilimigras et al. [58] reported stress-induced changes in behavior and the gut microbiota in mice, but there was no correlation between any of the gut microbiota changes with behavior changes. Conflicting results could be due to the site of microbiota sampling: Bangsgaard Bendtsen et al. [12] found that stress-induced behavioral changes correlated with cecal but not fecal microbiota changes.

Correlation of gut microbiota composition with behavior does not show causality. It is equally likely that differing changes in the gut due to different levels of perceived stress in stress-resilient or stress-sensitive individuals are the mediator for changes to the gut microbiota. However, intervention with prebiotic and probiotics have been able to alleviate stress-induced changes in emotional behavior. People given 30 days of a probiotic mix (*L. helveticus* R0052 and *B. longum* R0175) had reduced anxiety, depression, and perceived stress scores, as well as a decrease in 24 h urinary free cortisol from baseline concentrations [47]. The baseline anxiety and depression scores of the participants ranged from low to moderately high. In mice, oral supplementation with *Bifidobacterium* (LAC-B Granular Powder) increased the number of mice resilient to social defeat stress, and prevented a stress-induced decrease in sucrose intake [62]. *L. reuteri* 23272 given to male mice during chronic, mild stress decreased despair behavior in the forced swim test [15]. A probiotic (*L. rhamnosus* GG), prebiotic mix (polydextrose and galactooligosaccharide) or combined in a synbiotic mix, following maternal separation stress in male and female Sprague Dawley rats reduced stress-induced increases in anxiety-like behavior. The synbiotic had the greatest effect and was also able to ameliorate stress-induced memory changes [64]. An increase in stress-induced defecation was able to be prevented by prebiotic (fructooligosaccharides and galactooligosaccharides) supplementation [61].

It is possible that the positive action of probiotic and prebiotic supplementation on emotional behaviors may be more effective following stress. Sprague Dawley rats given a probiotic (*B. infantis* 35624) for 40 days following maternal deprivation stress, had reduced stress-induced immobility in the forced swim test [40] whereas the same daily dose of the same probiotic (although for only 14 days) without the stress intervention did not affect behavior [43]. Similarly, stress-induced increases in anxiety-like and depressive-like behaviors in mice were able to be ameliorated by dietary supplementation with prebiotics fructooligosaccharides and galactooligosaccharides [61]. Basal and acute stress-induced corticosterone levels were also reduced, and the prebiotic supplement prevented a stress-induced decrease in the *Actinobacteria*: *Proteobacteria* ratio and the relative abundance of *Bifidobacterium* and *Lactobacillus.* The decreased *Actinobacteria*: *Proteobacteria* ratio may have reflected a decrease in inflammation-reducing bacteria such as *Bifidobacterium* and an increase in opportunistic pathogens and bacteria with LPS. This may explain some of the stress-related changes in inflammation and mood. The prebiotic supplementation, given in a prior study with no stress intervention, had a much weaker effect on behavior [61]. Finally, a reduction in anxiety-like behavior was found in BALB/c stress-sensitive mice following supplementation of *L. rhamnosus* JB-1 [36], but the same probiotic given to healthy men did not alter HPA response or subjective mood or stress measures [44].

It is also possible that stress is one of the reasons why the results of animal intervention studies do not always translate well to human research. Laboratory conditions are neither reflective of real life for humans nor the animals involved in the research. Laboratory conditions can be stressful for animals, for example single housing [191]. This means that many intervention studies in animal may be effective by alleviating stress-induced changes in the gut-microbiota and/or physiology. If the same stress-induced changes are not present in human study participants then there may be no effect.

### Considerations for Future Research

Defining stress is not straightforward, and could have an impact on research results.

“Stress” is a broad term. External stress is defined by the environmental conditions, whereas perceived (internal) stress depends on how the individual feels. It is often assumed that under environmental stress all the individuals are also experiencing perceived stress but this may not be true. There is also a difference between how individuals experience perceived stress. “Good stress” is termed eustress and can improve performance and mood. “Bad stress” is what is typically considered to be stress and is sometimes defined as distress. In a similar vein, it is possible that animals in studies where stress is induced are experiencing environmental stress but not perceived stress. A careful definition of the type of stress being measured is important, and the measurement of baseline stress levels (e.g., corticosterone in hair or feces) as well as acute levels is beneficial.

The type of biological processes activated under stress also needs to be differentiated. HPA-axis activation may affect the MGBA differently than sympathetic nervous system activation. It is unclear whether the MGBA increases the risk of chronic perceived stress developing into anxiety and/or depression, or whether it increases the risk of an individual experiencing chronic perceived stress under high environmental stress. It may do both.

There is a reported association between diet and depression but the nature of the relationship is still unclear. Some research indicates that a healthy diet is protective against developing depression, but there are also many studies showing no effect [192]. Dietary manipulation of the gut microbiota composition may be a key variable in the diet-depression relationship, especially in preventing stress-induced changes in the gut microbiota [192]. Taylor, et al. [193] found independent relationships between the gut microbiota and mood (stress, anxiety and depression); and dietary factors and mood, with the microbiota-mood associations were mediated by fiber intake.

## 6. Conclusions

Stress-induced changes in the gut microbiota are a key variable which needs to be considered more in mood research. The limited research available suggests that promoting the growth of commensal bacteria, particularly those considered to be probiotic, is likely to confer some increase in emotional resilience under stress. Whether this is through direct prevention of effects from stress-altered gut microbiota, or through alleviation of physiological consequences of stress-induced changes, or both, is unknown. More research is needed. The mechanisms are still being elucidated and which individuals are more likely to respond to microbial support under stress, and whether there are ages, types of stress, time points in which interventions may be more or less effective, remain unknown. There is strong evidence for a role of lactobacillus, both as probiotic supplement or as commensal bacteria and research into probiotics and their mechanisms continues. The consequences of stress and interventions in different life periods, especially in early life should continue to be investigated. Dietary manipulation is another key area for research, particularly because diet will affect the gut microbiota composition and function. More nuanced physiological and psychological measurements are needed in order to differentiate between environmental, perceived stress and stress-related physiological processes.

## Figures and Tables

**Table 1:** Summary of studies testing the effects of probiotic supplements on mood. Effect on mood is indicated with: + for a positive effect on mood, − for a negative effect on mood, and / for no effect on mood. Abbreviations: LDB, Light Dark Box; SDT, Step Down Test; FST, Forced Swim Test; SPT, Sucrose Preference Test; PSS, Perceived Stress Scale; BAI, Beck Depression Inventory; BDI, Beck Anxiety Inventory; HADS, Hospital Anxiety and Depression scale; POMS, Profile of Mood State.

Subject, Study Design and Model	Probiotic	Dose and Administration	Treatment Duration	Effect on Mood		Reference
*Animal Studies*						
Male AKR mice with parasite-induced *(Trichuris muris*) chronic gastrointestinal inflammation	*Bifidobacterium longum* NCC3001 and *Lactobacillus rhamnosus* NCC4007	Gavaged daily, dose not specified	10 days	Reduction in anxiety-like behaviors in the LDB	+	[29]
Immunodeficient (B and T cell-deficient) male and female Rag 1^−/−^ mice	*L. rhamnosus* R0011 and *Lactobacillus helveticus* R0052	10^9^ CFU/mL in drinking water daily	4 weeks	Probiotic supplement normalized deficits in anxiety in LDB tests	+	[38]
Male C57BL/6 mice with liver inflammation-induced sickness behavior and brain inflammation	Commercial mixture VSL#3: *L. casei*, *L. plantarum*, *L. acidophilus and L. delbrueckii subsp. Bulgaricus*, *B. longum*, *B. breve and Bifidobacterium infantis*, *Streptococcus salivarius subsp. Thermophiles.* Strains unspecified	1.7 billion bacteria/day, gavaged daily	10 days	Prevention of a decrease in social interaction	+	[37]
Male AKR mice with chemically induced colitis	*B. longum* NCC3001 -	100 µL of 1 × 10^10^ CFU	7 days	A probiotic supplementation reduced anxiety-like behavior in SDT, but only when the vagus nerve was intact	+/	[39]
Male Sprague Dawley Rats	*B. bifidum* W23, *B. lactis* W52, *L. acidophilus* W37, *L. brevis* W63, *L. casei* W56, *L. salivarius* W24, *L. lactis* W19, *L. lactis* W58	4.5 g (2.5 × 10^9^ CFU/g) of freeze-dried powder in 30 mL of tap water per cage (2 rats) daily	10 weeks	A probiotic mix decreased depressive-like behavior in FST	+	[35]
Male Sprague Dawley rats following maternal separation stress	*B. infantis* 35624	1 × 10^10^ live bacterial cells/100 mL drinking water	55 days	A probiotic supplement ameriorated MSS induced depressive-like behavior in FST	+	[40]
A probiotic given alongside 3 weeks of restraint stress in male Sprague Dawley rats	*L. helveticus ns8*	10^9^ CFU/mL live bacteria in drinking water	3 weeks	Probiotic ameliorated stress-induced depressive-like behavior in SPT, and anxiety like behavior in EPM	+	[41]
Male BALB/c mice	*L. rhamnosus* JB-1	10^9^ CFU, gavaged daily	28 days	Decreased anxiety-like behaviors in the EPM	+	[36]
Male Sprague Dawley rats	*L. casei 54-2-33*	10^4^ CFU/mL in drinking water	14 days	Increase in anxiety-like behavior in the OFT and no difference in anxiety-like behavior in the EPM	−	[42]
Male Sprague Dawley rats	*B. infantis* 35624	1 × 10^10^ live bacterial cells/100 mL drinking water	14 days	No decrease in depressive-like behaviors in FST	/	[43]
*Human Studies*						
Healthy adult men	*L. rhamnosus* JB-1	10^9^ CFU, probiotic capsule, daily	8 weeks	No reduction in subjective stress measure, depression or anxiety scores on the PSS, BAI or BDI scales or improve cognitive measures	/	[44]
Healthy men and women	*L. helveticus* R0052 and *B. longum* R0175	3 × 10^9^ CFU probiotic capsule daily	30 days	Reduction in depression and anxiety scores (HADS). In a subset of people with low baseline urinary cortisol, the perceived stress scores were also reduced by the probiotic	+	[47,48]
Healthy men and women	*B. bifidum* W23, *B. lactis* W52, *L. acidophilus* W37, *L. brevis* W63, *L. casei* W56, *L. salivarius* W24, and *Lactococcus lactis* (W19 and W58)	2.5 × 10^9^ CFU probiotic capsule daily	4 weeks	Reduction in participant’s cognitive reactivity to sad mood	+	[49]
Men and women with chronic fatigue syndrome	*L. casei* Shirota	8 × 10^9^ CFU probiotic capsule daily	2 months	Improved anxiety (BAI) but not depressive (BDI) symptoms	+/	[50]
Healthy men and women	Milk drink containing probiotic *L. casei* Shirota	6.5 × 10^9^ CFU in a milk drink		Improvement in mood in POMS only in those who already had low mood	+/	[51]
Men and women with irritable bowel syndrome	Yohgurt containing *Lactobacillus paracasei*, ssp. paracasei F19, *L. acidophilus La5* and *B. lactis Bb12* (Cultura; active)	5 × 10^7^ cfu/mL × 200 mL milk drink, daily	8 weeks	The probiotic yoghurt drink did not improve mood scores in HADS	/	[45]
Men and women with diagnosed depression	*B. bifidum*, *L. acidophilus*, *and L. casei* (strains not specified)	*L. acidophilus* (2 × 10^9^ CFU/g), *L.casei* (2 × 10^9^ CFU/g), *B. bifidum* (2 × 10^9^ CFU/g), amount not specified	8 weeks	Reduction in symptoms of depression I BDI, along with fasting plasma insulin, glutathione, and C-reactive protein	+	[46]

**Table 2:** Summary of studies investigating links between stress and the gut microbiota. Microbiota which show an association (not necessarily causality) with stress-resilience and stress-sensitivity are indicated in the columns labeled SR and SS, respectively. This depends on the study but may mean, e.g., a probiotic supplement which increased stress-resilience, or an increase in relative abundance of the microbiota in stress-resilient individuals. Abbreviations: EPM, Elevated Plus Maze; FST, Forced Swim Test; TST, Tail Suspension Test; HbA1c, Hemoglobin A1c; IL, Interleukin; IFN-γ, Interferon gamma; LDB, Light-Dark Box; OFT, Open Field Test; NOR, Novel Object Recognition; MSS, Maternal Separation Stress.

Study Design (Stress, Subjects, Intervention)	Results	SR	SS	Reference
FST BALB/c mice (M, adult) Probiotic *Lactobacillus rhamnosus* JB-1, 28 days prior to FST/Vagotomy	Increase in anxiety-like (EPM) and depressive like behaviour (FST). Both ameliorated by probiotic Stress-induced increase in corticosterone ameliorated by probiotic. Stress-induced hyperthermia not affected by probiotic. Vagotomy prevented the anxiolytic effects of the probiotic. Changes in gut microbiota not reported	Probiotic: *L. rhamnosus* JB-1	Not applicable	[36]
Chronic mild stress, 7 w C57BL/6J Mice (M, 7 w) Probiotic: *Lactobacillus* reuteri ATCC 23272, 2 w during stress and 2 w post-stress	Increase in depression-like behaviour (FST): prevented by probiotic. Increase in serum kynurenine following stress. Prevented by probiotic. Inhibition of the enzyme IDO1 by *Lactobacillus*-produced reactive oxygen species (H_2_0_2_) in vivo Decreased (fecal) class *Bacillus*, specifically genera *Lactobacillus* and *Turicibacter*	Probiotic: *L. reuteri* ATCC 23272	Decrease in (fecal) *Lactobacillus*	[15]
Grid floor stress, 15 d BALB/c mice (F, 5 w) No intervention	Increase in anxiety-like (triple test) and depressive-like behaviour (TST) Lower blood glucose but highter HbA1c were found. Cytokines were reduced in the control mice but not the stressed mice. Correlations were found between IL-6, IFN-γ, and behavior in the LDB, EPM, and OFT. Increase in (cecal) *Odoribacter*, *Alistipes* and an unclassified genus from the *Coriobacteriaceae* family. *Lachnospiraceae* correlated to risk assessment behaviors. *Bacterioides* correlated with immobility in TST; *Ruminococccaceae* correlated to entries to closed arms in triple test (anxiety/activity)	Not applicable	*Bacterioides*; *Ruminococccaceae*	[12]
MSS, 15 d Sprague Dawley rats, sex not stated, 4 d neonatal Probiotic *L. rhamnosus* strain R0011 (95%) and *L. helveticus* strain R0052 (5%), 15 d during stress	Behaviour not measured Increase in serum cortisol and gut permeability. Prevented by probiotic Decrease in genus *Lactobacillus*. Increase in bacterial adherence and penetration into mucosal cells. Increases in cortisol, gut permeability, and bacterial adherence/penetration was prevented by probiotic supplementation.	Probiotic: *L. rhamnosus* strain R0011 (95%) and *Lactobacillus helveticus strain* R0052 (5%)	Not applicable	[59]
MSS, 15 d Sprague Dawley rats (M, 90 d) Probiotic *Bifidobacterium* infantis 35624, 45 days	Increase in depressive-like behviour (FST), ameliorated by probiotic supplementation No difference in plasma corticosterone, L-kynurenine, tryptophan or kynurenic acid. An increase in IL-6 following stimulation with immune stimulant concanavalin A was prevented by the probiotic Gut microbiota not measured	Probiotic: *B. infantis* 35624	Not applicable	[40].
MSS, 10 d Sprague Dawley rats (M, 7–8 w) No intervention	Increased stress-induced faecal boli number in the OFT, but no changes in behavior Increased plasma corticosterone and increased systemic immune response in response to in vitro LPS challenge. Decreased pain threshold Change in microbiota structure (taxa not specified)	Not applicable	Not applicable	[16]
MSS, 1 w Infant rhesus monkeys (M+F, 6–9 m) No intervention	Decrease in total abundance of fecal bacteria and *Lactobacillus* by day 3, but back to normal after a week	Not applicable	Not applicable	[67]
Prenatal stress Human infants No intervention	Gastrointestinal symptoms more common in babies from mothers who reported higher stress. Cortisol and stress related questionnaires did not correlate in the mothers. Increased fecal *Escherichia-enterobacteria* and lower lactic acid bacteria and *Actinobacteria*	Not applicable	Not applicable	[69]
Prenatal (dam exposed to CMS) Offspring of pregnant C57BL mice exposed to CMS stress No intervention	Behavior not measured Increased fecal *Rikenellaceae* and *Odoribacter*, *Mucispirillum* and a decrease in *Bacteroides*	Not applicable	Not applicable	[66]
Prenatal (dam exposed to restraint stress) Offspring of pregnant C57/B16 mice No intervention	Offspring showed increase in anxiey-like behvior (EPM, NOR) in adulthood Increased plasma IL-1β in placenta and fetal brains but did not persist till adulthood. Decreased BDNF found in maternal placenta and in brains of adult offspring. Microbial community composition clustered differently in the stress group from control in both pregnant dams and their offspring	Not applicable	Not applicable	[65]
Restraint Stress (16 h/d × 7 d) Swiss Webster & CD-1 mice (M, 6–8 w) Fecal transplant from stressed mice to germ free mice	Behaviour not measured Increased inflammatory response to colonic pathogen in germ free mice with fecal transplant from stressor exposed mice. Increased (fecal) *Firmicutes* and decreased *Actinobacteria* and *Bifidobacterium*.	Not applicable	Not applicable	[63]
Restraint Stress (6 h/d × 3 w) Sprague Dawley rats (M, 220–240 g) Probiotic: *L. helveticus* ns8, 26 d	Increased depressive-like behavior (SPT) and anxiety-like behavior (EPM, OF). Body weight was reduced. Increase in plasma corticosterone and pro-inflammatory cytokines TNF-α and IFN-γ and decrease in plasma IL-10. Decreased BDNF in the hippocampus, prevented by probiotic Stress-induced changes in behaviour, corticosterone, IL-10, BDNF were prevented by the probiotic supplementation Gut microbiota not measured	Probiotic: *L. helveticus* ns8	Not applicable	[41]
Restraint stress, 12 h/d × 7 d CD1 mice (M, 8 w) No intervention	Behaviour not measured Increased TNF-α gene expression in colonic tissue Total bacteria and Gram negative bacteria increased in small intestine, cecum, and large intestine. Decrease in bacterial diversity and richness. Reduced family *Porphyromonadaceae*, specifically genus *Tannerella*. Increased colonization by introduced pathogen *Citrobacter rodentium*	Not applicable	Not applicable	[60]
Restraint Stress, 15 h/d × 7 d CD-1 mice (M, 6–8 w) No intervention	In the (colonic) mucosca-associated bacteria, a decrease in the families *S24-7* and *Lactobacillaceae* and genera *Lactobacillus* spp., were found, and in an increase in the family *Ruminococcaceae*, and genera *Oscillospira*. In the luminal bacteria, a decrease in the family *S24-7*, as well as genera *Adlercreutzia*, and an unclassified genus in *S24-7* were found	Not applicable	Not applicable	[13]
Restraint stress/FST alternated, 19 days CF-1 mice (M+F, 6 w)	Distance travelled in EPM and LDB increased, increased rearings in OF. Blood collected after behavioral tests Males had higher corticosterone levels following acute stress (behavioral testing) Increase in family *Lachnospiraceae*. Decrease in genus *Sarcina* only in females. *Ruminococcus* gnavus increased in females but decreased in males	Not applicable	Not applicable	[58]
Social stress: cage in cage aggressor CD1 and C57BL/6 mice (M, 6–8 w)	Behaviour not measured No difference in colonic cytokines Decrease in relative abundance of families *Porphyromonadaceae* and *Lactobacilliaceae*, and genera *Lactobacillus*, *Parabacteroides*, and an unclassified genus from phylum *Firmicutes* and unclassified genus from class *Bacilli*. The absolute abundance of *lactobacilli* was also reduced, specifically *L. reuteri*, but only in the outbred CD-1, not the inbred C57BL/6 mice	Not applicable	Not applicable	[56]
Social stress: chronic social defeat C57BL/6 mice (M, 8 w)	Decrease in social interaction Increase in (fecal) genus *Bifidobacterium* in the stress resilient group. Not detected in the control group or stress-sensitive group	*Bifidobacterium*	Not applicable	[62]
Social stress: resident intruder, 6 h/d × 10 d C57BL/6J male, juvenile (5–6 wk)	Behaviour not measured Differed across time points. Key changes were a decrease in phylums Bacteroidetes, Firmicutes, Verrucomicrobia; and genera Oscilospira and Anaeroplasma, with a trend in decrease in Lactobacillus. An increase and decrease in Akkermansia were found at different time points. A trend of increase in phylum Proteobacteria was found	Not applicable	Not applicable	[57]
Social stressor (6 d × 2 h/d) CD1 Mice (M, 8 w) Antibiotics (ampicillin (1 mg/mL), vancomycin (0.5 mg/mL), neomycin sulfate (1 mg/mL), and metronidazole (1 mg/mL))	Behaviour not measured Increase in proinflammatory markers, particularly IL-6, prevented in antibiotic group Immediately after induced stress, the (cecal) microbiome of mice had consistently altered within the group, and clustered separately from the control group, but after 15 h, the separation was no longer as clear, with variation within the stress group.Within genera, decrease in *Bacteroides*,increase in *Clostridium*, trend of decrease in *Lactobacillus* Stress-induced increases in plasma IL-6 was inversley correlated with relative abundances of genera *Coprococcus*, *Pseudobutyrivibrio* and positively correlated with *Dorea*	Not applicable	Not applicable	[11]
Water Avoidance Stress (1 h/d × 7 d) C57BL/6N mice (F, 6 w) Antibiotics during stress (Bacitracin A, Neomycin, Amphotericin B)	Pain related behavior in response to intracolonic capsaicum increased. Slightly mitigated with antibiotics. Increased fecal pellet output, plasma corticosterone, and adrenal gland weight. Increased luminal s-IgA levels. In the colon tissue, cannabanoid receptors increased marginally, and tryptophan hydroxylase (TPH1) expression increased by 40% Antibiotics and stress enhanced bacterial adherence to luminal wall. Fecal *Clostridium* coccoideds cluster XIVa was increased, and *Verrucobactera*, *Lactobacillus* and *Enterococcus* spp. decreased	Not applicable	Not applicable	[190]
